# *EML4*-*ALK*融合基因在非小细胞肺癌中的临床意义

**DOI:** 10.3779/j.issn.1009-3419.2013.02.07

**Published:** 2013-02-20

**Affiliations:** 233000 蚌埠，蚌埠医学院第一附属医院肿瘤内科 Department of Medical Oncology, the First Affiliated Hospital of Bengbu Medical College, Bengbu 233000, China

**Keywords:** 肺肿瘤, *EML4*-*ALK*融合基因, 分子靶向治疗, Lung neoplasms, *EML4*-*ALK*, Molecular targeted therapy

## Abstract

世界范围内肺癌位居所有癌症致死的第一位，其中大多数为非小细胞肺癌（non-small cell lung cancer, NSCLC）。目前，分子靶向治疗是NSCLC的治疗中最有发展前景的部分。近年来，NSCLC新的分子生物靶点例如棘皮动物微管相关类蛋白4与间变性淋巴瘤激酶融合基因越来越受到关注。本文旨在介绍*EML4*-*ALK*融合基因的基本结构、临床病理学特征、检测方法、ALK抑制剂及其在NSCLC治疗中的意义。

## Abstract (en)

Lung cancer continues to be the leading cause of cancer death worldwide, and non-small cell lung cancer (NSCLC) is the most common type of lung cancer. Molecular targeted therapy is the most promising development part in the treatment of NSCLC. In recent years, more and more attention has been focused on some new molecular targets in NSCLC, such as echinodem microtubule-associated protein-like4/anaplastic lymphoma kinase (*EML4*-*ALK*) fusion gene. This review will focus on the basic structure, clinicopathologic features, detection method, ALK inhibitor and therapeutic meaning of *EML4*-*ALK* in NSCLC.

肺癌是世界范围内最常见的恶性肿瘤之一，也是发病率及癌症相关致死率最高的恶性肿瘤^[1]^。非小细胞肺癌（non-small cell lung cancer, NSCLC）占肺癌的80%-85%。由于早期症状不明显，多数患者确诊时多为中晚期，传统的治疗方法如手术、放疗、化疗能改善近期疗效，但对生存期的延长作用有限。分子靶向治疗是近年来恶性肿瘤治疗领域的“王牌”，其特点是能选择性地作用于肿瘤细胞，定向阻断癌细胞的转移、增殖、信号传导，阻止肿瘤新生血管的生成，破坏癌细胞的新陈代谢^[2]^，故其特异性高，不良反应小，是目前最具前景的研究领域。棘皮动物微管相关类蛋白4与间变性淋巴瘤激酶融合基因（chinodem microtubule-associated protein-like 4/anaplastic lymphoma kinase, *EML4*-*ALK*）有望成为继表皮生长因子受体（epidermal growth factor receptor, EGFR）后另一种有明确疗效预测作用的分子标志物，它被证实是NSCLC发生发展过程中独立而又关键的分子靶点，在NSCLC中的发生率约为5%。本文主要介绍*EML4*-*ALK*融合基因的结构特点、临床病理特征，常用于筛检*EML4*-*ALK*融合基因的方法，简述*ALK*抑制剂临床研究进展，并评价其在NSCLC治疗中的地位和意义。

## 1 *EML4*-*ALK*融合基因概述

*ALK*基因最初是在间变性T细胞淋巴瘤和炎性成纤维细胞瘤中发现。目前已发现其参与形成的融合基因（*X*-*ALK*）与多种肿瘤的发生密切相关^[3]^。2007年Soda等^[4]^首次发现*EML4*-*ALK*融合基因存在于部分NSCLC中。研究者在1例男性吸烟的肺腺癌患者标本中扩增出由*EML4*和*ALK*融合而成的DNA片段，它编码的蛋白质包含了1, 059个氨基酸。蛋白质的N端（残基1-496）为人*EML4*基因的一部分，而C端残基（497-1, 059）则是人*ALK*基因的胞内域。

EML4隶属于棘皮动物微管相关类蛋白家族，包括WD-重复区、N端Basic区和HELP域。它们都和EML4-ALK肿瘤生成的潜能有关，且Basic区在EML4-ALK二聚体化的过程中扮演着重要的角色，去除Basic区可使EML4-ALK融合蛋白的催化活性降低84%，可以推断Basic区可能是EML4-ALK融合蛋白发挥作用的关键区域。

ALK则是胰岛素受体超家族的成员，可与*ATIC*-、*TFG*-、*CLTC*-等多种基因发生融合，其结构包括：胞外配体结合区、跨膜区、胞内酪氨酸激酶区。

EML4与ALK定位于2号染色体短臂上，但两者方向相反，相隔12 Mb，融合时须EML4或ALK其中之一反向与对方连接，*EML4*-*ALK*基因亚型取决于两者断裂相接的位置，因此EML4断裂点呈现出多变性，目前已检测到的断裂点有2、6、13、14、15、17、18、20号外显子，这些形成了至少11种*EML4*-*ALK*融合基因亚型，已有实验^[4-11]^证实它们中的大部分能促进肿瘤生成。

## 2 *EML4*-*ALK*融合基因的检测方法

检测*EML4*-*ALK*融合基因的方法很多，主要包括免疫组织化学、逆转录聚合酶链式反应、荧光原位杂交技术和RACE-偶联PCR测序等，现将常用的检测方法简述如下：

### 2.1 免疫组织化学（immunohistochemistry, IHC）

IHC是病理检测的主要方法之一。因其操作简便、成本低、工作量较小而被广泛应用于临床和科研工作中，IHC的最大优势是能检测到肿瘤特异性抗原，但不会破坏细胞和组织的结构特征。实验证实IHC同FISH和RT-PCR检测*EML4*-*ALK*融合基因的结果具有一致性，但其敏感性劣于FISH和RT-PCR法，而且不能鉴定ALK融合患者具体来自哪种变体亚型。因此，可以将IHC法作为筛查*EML4*-*ALK*融合基因的方法。

### 2.2 逆转录聚合酶链式反应（reverse transcription-polymerase chain reaction, RT-PCR）

RT-PCR法是一种敏感性高的基因检测方法，可以检测到很低拷贝数的RNA，可能是确认NSCLC存在*ALK*融合基因的一种快速诊断方法。利用术后或其它方法取得的标本，获得总RNA后进行RT-PCR，对PCR产物进一步分析以确定是否含有*EML4*-*ALK*融合基因，该法需要高质量的RNA，但石蜡切片中的DNA及RNA在检测过程中会逐渐降解，最终会影响检测结果的准确性，因此，RT-PCR更适用于新鲜组织标本的基因检测。但其缺点在于对引物要求很高；分辨不出*EML4*-*ALK*融合基因的未知亚型；假阳性率较高。由于此方法操作复杂，在常规临床诊断实验室可能难于施行。

### 2.3 荧光原位杂交（fluorescence *in situ* hybridization, FISH）

FISH法应用探针特异性标记细胞核中ALK断裂点来检测ALK重排，分离的荧光信号提示存在ALK重排。相比IHC和RT-PCR，FISH法的敏感性和特异性都很高，但并不是万无一失。与PCR不同，FISH不能鉴别出不同的*EML4*-*ALK*融合基因变异体，且其价格昂贵，分离的荧光信号不易解释。目前，ALK靶向药物克里唑替尼（Crizotinib, PF-02341066）临床前试验中将FISH法作为*EML4*-*ALK*融合基因的检测方法。

### 2.4 RACE-偶联PCR测序（RACE-coupled PCR sequencing）

张绪超等^[12]^使用该法检测分析*ALK*基因的融合变异，他们采用cDNA末端快速扩增（PACE）技术结合两轮PCP技术来扩增*ALK*基因的融合变体，敏感性高，其优点是能检测到*EML4*与*ALK*或其它任何基因的融合，测序后还能发现是来自哪一种EML4-ALK变体的融合。但目前只有张绪超等^[12]^的研究应用了这种检测技术，还需要更多的研究加以验证。

越来越多的标本进行了*EML4*-*ALK*融合基因的检测，其中以RT-PCR法的应用最为广泛，但目前仍无敏感特异的方法用于*EML4*-*ALK*融合基因的检测。未来的发展方向在于寻找有效可行的方法用于准确检测*ALK*基因融合。

## 3 *EML4*-*ALK*融合基因与NSCLC的临床病理关系

表皮生长因子受体酪氨酸激酶抑制剂（EGFR-tyrosine kinase inhibitor, EGFR-TKI）治疗有效的患者被证实有其独特的临床病理学特征，如：亚裔、不吸烟、女性、腺癌。决定EGFR-TKI疗效的关键在于EGFR的19、21外显子突变。NSCLC中*EML4*-*ALK*融合基因阳性的患者是否也有其独特的临床病理特征，世界各地的科学家们对此进行了深入的研究。

日本的Inamura等^[13]^用RT-PCR法检测了363例肺癌患者的病理标本，11例（3%）标本中检出*EML4*-*ALK*融合基因，患者病理分型均为腺癌，进一步研究证实*EML4*-*ALK*融合基因阳性的NSCLC具有以下的特征：腺泡组织来源，分化更差；常有TTF-1表达，偶有*TP53*突变，极少有*EGFR*及*K*-*ras*的突变；更常见于轻度或不吸烟的患者。

香港的Wong等^[8]^检测了266例原发性肺癌患者的术后病理标本，对*EML4*-*ALK*、*KRAS*、*EGFR* 3种基因的阳性率进行比较和统计学分析处理，共检出野生型EGFR、KRAS阳性119例，其中13例（4.9%）*EML4*-*ALK*基因表达阳性患者，差异有统计学意义，这13例包括11例腺癌和2例腺鳞癌，他们也发现了*EML4*-*ALK*融合基因更常见于不吸烟、年轻和EGFR、K-ras野生型的肺癌患者中，其差异具有统计学意义。

根据以上试验结果，2009年Shaw等^[14]^进行了更有针对性的研究，共选择了141例NSCLC患者，入组条件为女性、亚裔、不吸烟或轻度吸烟和腺癌中至少有2项符合，用FISH方法检测了标本中的情况，共检出了19例（13%）*EML4*-*ALK*融合基因，包括18例腺癌和1例腺鳞癌，发生*EML4*-*ALK*、*EGFR*及*K*-*ras*突变的患者没有重叠。

2009年张绪超等^[15]^用RACE-PCR在109例NSCLC的标本中检出12例EML4-ALK阳性（11.01%）。所有*EML4*-*ALK*融合基因阳性的NSCLC患者均不与*K*-*ras*突变共存，但意外的在1例女性肺腺癌患者中发现了*EML4*-*ALK*融合基因与*EGFR* 19号外显子突变的共存。

多项临床研究已经证实：*EML4*-*ALK*融合基因是NSCLC患者又一常见的临床亚组，其典型的临床特征包括：男性、既往不吸烟或轻度吸烟史、年轻患者、腺癌或以腺癌细胞为主的病理类型、*EML4*-*ALK*融合基因与*EGFR*突变和*K*-*ras*突变不共存。这对临床上可以更加快速有效地筛选目标人群有着极大的意义。

## 4 ALK抑制剂

EML4-ALK作为一个潜在的NSCLC的治疗靶点，目前已进入临床实验研究阶段的ALK抑制剂只有克里唑替尼（PF-02341066），它同时也是MET/HGF受体酪氨酸激酶抑制剂，由辉瑞公司研制。它是一种选择性的ATP竟争性小分子抑制剂，抑制c-Met/肝细胞生长因子受体（hepatocyte growth factor receptor, HGFR）和ALK酪氨酸激酶及他们的致癌变异体（如c-Met/HGFR突变变异体或ALK融合蛋白）。体内外试验均证实，此药可以剂量依赖性抑制肿瘤细胞c-Met/HGFR、ALK和某些变异体的磷酸化及激酶靶位依赖功能。推荐剂量为250 mg（po, Bid）。

Ⅰ期临床试验结果^[16]^显示，在82例可评价疗效的患者中客观缓解率为57%，8周疾病控制率达90%。平均治疗时间是6.4个月，其中1例完全缓解，46例部分缓解，27例疾病稳定。6个月无进展生存率为72%，中位无进展生存期9.2个月。所有患者MET为阴性（克里唑替尼的另外一个靶点），表明治疗作用是通过抑制ALK完成。轻微的胃肠道反应为主要不良反应，如恶心（54%）、腹泻（48%）和呕吐（44%），但患者完全可以耐受，3/4度毒性只见于谷丙和谷草转氨酶的升高。

Ⅱ期临床试验^[17]^证实，在50例可评价疗效的患者中，客观缓解率为64%，8周疾病控制率达到了90%，6个月无进展生存率为72%，且中位治疗时间均已多于25.5周，生存终点尚在观察之中。胃肠道反应仍是克里唑替尼的主要副反应，但患者均可耐受。

克里唑替尼的Ⅰ期/Ⅱ期临床试验结果均证实了该药抗癌效果满意，安全性好。因此，2011年8月26日美国食品药品管理局批准克里唑替尼（PF-02341066）直接进入Ⅲ期临床试验（Profile-1007），中国也参与其中，Profile-1007是克里唑替尼的首个Ⅲ期临床试验，在2012年欧洲肿瘤内科学会（European Society for Medical Oncology, ESMO）上，Shaw等^[18]^报告了该试验的中期结果，该试验共入组了347例既往接受过含铂一线化疗方案的Ⅲb期/Ⅳ期NSCLC患者，所有患者均经FISH证实ALK阳性，实验组的173例患者给予克里唑替尼（250 mg bid, q 21d），对照组的174例患者分别给予多西他赛（75 mg/m^2^, q 21d）或培美曲塞（500 mg/m^2^, q 21d）。随机接受单药化疗的患者在疾病进展后可接受克里唑替尼治疗，因此，化疗组的大多数患者最终接受了克里唑替尼治疗。两组总有效率分别为65.3%和19.5%（OR=3.4, 95%CI: 2.5-4.7, *P*＜0.001），两组无进展生存期为7.7个月和3.0个月（HR=0.49, 95%CI: 0.37-0.64, *P*＜0.000, 1）。以上试验结果证实，克里唑替尼相对于单药化疗组明显延长了无进展生存期，提高了客观缓解率。因此，对于*ALK*突变阳性的NSCLC患者来说，克里唑替尼可以作为晚期肺癌患者的标准治疗。Profile-1007比较克里唑替尼与二线化疗方案的疗效，而另一个Ⅲ期临床试验Profile-1014则比较的是克里唑替尼与一线化疗方案。该实验入组标准是既往没有接受过治疗的ALK阳性局部晚期/转移的非鳞状NSCLC患者，入组的334例患者被随机分配到克里唑替尼组和培美曲塞/顺铂或培美曲塞/卡铂组，主要观察终点是无进展生存期。另外一个单臂的Ⅱ期临床研究（Profile-1005）^[16]^入组了250例由于接受过多的治疗而不能入组Ⅲ期临床研究或者Ⅲ期临床研究中接受化疗后却进展的NSCLC患者，服用克里唑替尼观察其疗效。

在治疗初期克里唑替尼能取得非常好的疗效，但与EGFR-TKIs及其它TKI一样，最终肿瘤也会对克里唑替尼产生耐药。Choi等^[19]^报道了1例应用克里唑替尼治疗有效5个月后却发生继发耐药的病例。患者为28岁无吸烟史的男性肺腺癌，检测未发现*EGFR*突变，经过6周期的常规化疗后肿瘤出现了进展，检测发现肿瘤表达*EML4*-*ALK*融合基因的亚型1。经过1周的克里唑替尼治疗后，患者的症状较前明显好转。但5个月后肿瘤出现了进展，导致双肺肿瘤和胸腔积液的迅速增长。在该患者的胸水样本中发现EML4-ALK激酶域有两个突变（野生型ALK的cDNA核甘酸4 374和4 493，分别发生G→A和C→A的改变，频率分别为41.8%和14.0%），这两处碱基的改变导致该位点氨基酸改变，分别是C1156Y和L1196M，作者进一步通过细胞株试验及磷酸化检测验证了这两个点突变的确可以导致对克里唑替尼的耐药。针对L1196M耐药后的治疗，Katayama等^[20]^用比克里唑替尼活性更强的ALK抑制剂如TAE684和AP26113或是HSP90抑制剂进行治疗。Sakamoto等^[21]^也证实了ALK抑制剂CH5424802能高选择性的抑制L1196M突变的细胞生长。

## 5 小结及展望

目前认为，*EML4*-*ALK*融合基因是NSCLC新一类的亚型。*EML4*-*ALK*融合基因的形成是由2号染色体短臂插入引起的，它至少有11种亚型。NSCLC中*EML4*-*ALK*融合基因阳性更常见年轻男性患者、轻度吸烟∕不吸烟、腺癌、*K*-*ras*或*EGFR*野生型。常用来检测EML4-ALK的方法有IHC、RT-PCR、FISH和RACE-偶联PCR测序，各方法有其优缺点，有一定的互补性。ALK抑制剂克里唑替尼治疗EML4-ALK阳性的NSCLC能取得较佳的疗效，有望成为晚期NSCLC患者的标准治疗。

目前，基因检测已经从实验室走进了临床工作中，众多生物标志物的发现，为实现真正意义上的NSCLC“个体化”治疗提供了契机。建议在选择使用靶向治疗前，制定合理的生物标志物筛选方案，这有助于改善疗效，提高治疗成功率，避免某些过度治疗，节约医疗成本。以生物标志物为指导的个体化综合治疗模式将进一步提高NSCLC患者靶向治疗的疗效和患者的生存质量。

尽管克里唑替尼用于NSCLC患者的Ⅰ期、Ⅱ期临床试验结果令人鼓舞，但仍有许多问题有待进一步研究，如检测*EML4*-*ALK*融合基因的金标准是什么；*EGFR*突变与*EML4*-*ALK*融合基因的相互关系是什么；*EML4*-*ALK*融合基因的不同变体是否代表不同的临床意义；*EML4*-*ALK*融合基因的在NSCLC中的阳性率是否存在地理或种族差异；*EML4*-*ALK*融合基因是否确实是克里唑替尼治疗有效的疗效预测分子；与现有的治疗手段相比，克里唑替尼是否能取得明显的客观疗效和生存期优势；对共存型NSCLC患者而言，多靶点抑制剂会不会是更好的选择；耐药机制仍不清楚以及耐药后患者又该如何进行治疗等。期待更加深入的实验和临床研究来解决上述问题，从而使NSCLC的个性化治疗得以在基因水平上实现。
